# Alignment of Non-Covalent Interactions at Protein-Protein Interfaces

**DOI:** 10.1371/journal.pone.0001926

**Published:** 2008-04-02

**Authors:** Hongbo Zhu, Ingolf Sommer, Thomas Lengauer, Francisco S. Domingues

**Affiliations:** Max-Planck-Institut für Informatik, Saarbrücken, Germany; Vanderbilt University, United States of America

## Abstract

**Background:**

The study and comparison of protein-protein interfaces is essential for the understanding of the mechanisms of interaction between proteins. While there are many methods for comparing protein structures and protein binding sites, so far no methods have been reported for comparing the geometry of non-covalent interactions occurring at protein-protein interfaces.

**Methodology/Principal Findings:**

Here we present a method for aligning non-covalent interactions between different protein-protein interfaces. The method aligns the vector representations of van der Waals interactions and hydrogen bonds based on their geometry. The method has been applied to a dataset which comprises a variety of protein-protein interfaces. The alignments are consistent to a large extent with the results obtained using two other complementary approaches. In addition, we apply the method to three examples of protein mimicry. The method successfully aligns respective interfaces and allows for recognizing conserved interface regions.

**Conclusions/Significance:**

The Galinter method has been validated in the comparison of interfaces in which homologous subunits are involved, including cases of mimicry. The method is also applicable to comparing interfaces involving non-peptidic compounds. Galinter assists users in identifying local interface regions with similar patterns of non-covalent interactions. This is particularly relevant to the investigation of the molecular basis of interaction mimicry.

## Introduction

Protein-protein interactions are involved in most cellular processes as many proteins carry out their functions by forming complexes. These protein complexes consist of interacting polypeptide chains (subunits). The interfaces in such complexes are composed of complementary binding sites from the respective subunits.

The characterization of protein interfaces provides insights into protein interaction mechanisms. Such analysis is expected to have an impact on the prediction of interaction partners, as well as to assist in the design and engineering of protein interactions and interaction inhibitors. The physico-chemical properties of protein-protein interfaces have been previously investigated [1]–[4]. Interactions between proteins have been classified according to different criteria; in a review, Nooren and Thornton use the criteria composition, affinity, and lifetime to classify interactions as homo or hetero, obligate or non-obligate, and permanent or transient, respectively [5]. Methods have been developed for distinguishing different interaction types based on interface properties [6]–[8].

Detailed comparison of protein-protein interfaces is fundamental for their better characterization and for structure-based classification of protein complexes. With an increasing amount of structural models for protein complexes available in the Protein Data Bank (PDB) [9], protein complexes can now be compared systematically at the structural level. The structure similarity of protein complexes may be assessed at two levels: the similarity of the orientation of the binding sites relative to the folds of the subunits, and the local structure similarity of interfaces, as detailed in the next two paragraphs.

In a comprehensive study, Aloy *et al.* have analyzed the relationship between protein sequence similarity and the spatial orientation of protein interaction [10]. They discovered that among proteins with high sequence similarities the orientation of protein interaction tends to be conserved. Kim and colleagues have put forward a method for objectively comparing the orientations of interacting domains in two complexes [11]. They have divided protein domain-domain interfaces into different groups (face types), resulting in SCOPPI, a structural classification of protein-protein interfaces [12]. They have shown that similar protein domains may interact with distinct partners (non-homologous structures) using similar face types, but similar domains might also interact via different face types. Recently, using a similar method, Henschel *et al.* have identified cases of protein interaction mimicry, meaning that homologous subunits interact with non-homologous partners in the same relative orientation [13].

Local structure comparison of interfaces has been the focus of several other studies. Nussinov and colleagues have clustered all known protein-protein interfaces in the PDB by comparing the binding site C_α_ atoms using a geometric hashing procedure [14], [15]. Based on the analysis of the resulting clusters, they observed that proteins with different folds and functions may associate to yield interfaces of similar local structures [16]. Shulman-Peleg *et al.* have developed I2I-SiteEngine and MAPPIS, programs that compare and align the functional groups at a pair or set of interacting binding sites using a geometric hashing algorithm [17]–[19]. Similar methods have been developed for comparing protein binding sites for small molecules [20], [21], and they have been recently reviewed [22].

Protein complexes are stabilized by non-covalent interactions formed across interfaces (when we speak of non-covalent interaction we mean interactions between specific functional groups; when we speak of interaction, in general, we mean interactions between whole proteins composed of many non-covalent interactions). Non-covalent interactions at protein-protein or protein-ligand interfaces are often compared in order to characterize binding modes and to identify detailed structural differences. Biswal and colleagues have manually examined van der Waals (vdW) interactions and hydrogen bonds at two interfaces corresponding to a polymerase binding to two different inhibitors [23]. Deng *et al.* have represented interactions at a protein-ligand interface as a one-dimensional fingerprint descriptor for studying different docking results on the same protein [24]. Swint-Kruse has compared the interfaces of dimeric LacI complexes in distinct functional states [25]. The differences in fine structures of the interfaces have been identified by representing the set of non-covalent interactions as two-dimensional networks formed between interface residues [26]. Recently, Keskin and Nussinov have shown that proteins may interact with variable partners via collections of structurally conserved non-covalent interactions [27]. All of the above approaches require pre-computed sequence alignments or structure-based alignments of backbone atoms, and do not directly align the non-covalent interactions according to their conserved geometry.

Here, we present a novel method, Galinter, for aligning protein-protein interfaces. To our knowledge, this is the first method for explicitly comparing the geometry of non-covalent interactions at interfaces. The explicit comparison of non-covalent interactions provides an intuitive method of comparative analysis and visualization of binding modes, and for investigating the degree of conservation between interfaces. We have tested Galinter on a published dataset of interfaces, and have also applied the method to analyzing three medically relevant cases of protein mimicry.

## Methods

### Method workflow

**Figure 1:**
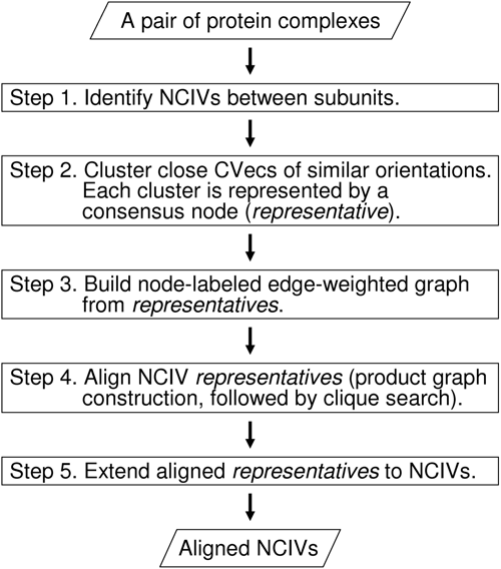
Flow chart of Galinter. (NCIV: non-covalent interaction vector; CVec: contact vector)

In this study, two types of non-covalent interactions are considered: van der Waals interactions and hydrogen bonds. These non-covalent interactions are represented as vectors (NCIVs) connecting the centers of two interacting atoms. The goal of the method is to find the largest set of NCIVs that can be superposed (structurally aligned) in similar geometric orientations. Two NCIVs (each from one interface) are matched in the alignment if they represent the same type of non-covalent interactions, and have similar distances and relative orientations to the other matched NCIVs within the respective interfaces. A graph-based method is applied for aligning NCIVs. The complete procedure is implemented in Galinter (Graph-based alignment of protein-protein interfaces). The workflow of the method is composed of the following five steps. Figure 1 provides a schematic overview.

#### Identifying NCIVs

For two protein complexes with known structures, two types of NCIVs between the interacting proteins are distinguished. Contact vectors (CVecs) are detected based on a distance criterion and represent van der Waals interactions. A CVec connects two heavy atoms if the distance between them is less than the sum of their respective van der Waals radii plus 1.0 Å. The user specifies one of the two binding sites as the head site and the other as the tail site. All CVecs point from the tail to the head site. Hydrogen bond vectors (HVecs) are the second type of NCIV. These are determined by adding hydrogen atoms to the protein structures with the REDUCE program [28] and by then applying a set of geometric criteria [29, see Figure S1 in the supplement]. The directions of the HVecs encode the hydrogen bonding donor-acceptor direction. The distance between a pair of NCIVs is defined as the Euclidean distance between the middle points of the head and tail points of the two vectors, respectively.

#### Clustering NCIVs

In this step, two CVecs are grouped into the same cluster if they are closer than 2.0 Å and if the angle between their orientations is at most 45°. Subsequently, a consensus vector is computed and then used as representative for each cluster. A complete linkage hierarchical clustering algorithm is employed to cluster the NCIVs. HVecs are not clustered and are directly taken as representatives. The distance between representatives is defined in the same way as the distance between NCIVs.

This clustering step is based on the observation that often there are small groups (size of 2–4) of CVecs with similar orientations (angle difference at most 45°). Clustering NCIVs also reduces the size of the alignment problem and enables Galinter to obtain results in reasonable run time (within minutes).

#### Generating a graph representation for protein-protein interfaces

In this step, each protein-protein interface is modeled as an undirected node-labeled edge-labeled graph *G*(*V*,*E*). Node set *V* consists of all the NCIV representatives obtained in the previous step. Each node is labeled as either a CVec representative, or a HVec representative. Two nodes *u*, *v* are connected by an edge if the distance between the corresponding NCIVs is in the range from 2.0 to 40.0 Å. Each edge is labeled with a 5-tuple *EdgeLabel*. In every *EdgeLabel*, the first value is the distance between the corresponding NCIVs, and the other four values are the distances between each pair of endpoints of these two NCIVs. We have chosen 2.0 Å as lower bound because in the previous clustering step the cluster radius is also 2.0 Å. The upper bound of 40.0 Å excludes less than 5% of the NCIVs, since more than 95% of the distances between all CVecs in a structurally non-redundant dataset [15] are at most 40.0 Å (data not shown).

#### Aligning representatives

Given two graphs *G*_1_(*V*_1_,*E*_1_) and *G*_2_(*V*_2_,*E*_2_) representing two protein-protein interfaces, the goal is to find all maximum common subgraphs *H*_1_ and *H*_2_ such that i) *H*_1_⊆*G*_1_, *H*_2_⊆*G*_2_, *H*_1_ and *H*_2_ are isomorphic *H*_1_≡*H*_2_, and ii) there is no pair (*H*_1_′, *H*_2_′) and such that *H*_1_⊆*H*_1_′⊆*G*_1_, *H*_2_⊆*H*_2_′⊆*G*_2_, *H*_1_′≡*H*_2_′, and *H*_1_′, *H*_2_′ have more nodes than *H*_1_ and *H*_2_, respectively.

The maximum common subgraph problem is transformed to the maximum clique problem in the traditional fashion [30], [31]. Maximal common subgraphs in *G*_1_ and *G*_2_ are identified by searching for maximal cliques in a product graph of *G*_1_ and *G*_2_ [31], [32]. The product graph *P*(*V_P_*,*E_P_*) has a node set *V_P_* = {(*u*_1_,*u*_2_) | *V*_1_×*V*_2_ and *label*(*u*_1_) = *label*(*u*_2_)}. In *P*, two nodes (*u*_1_,*u*_2_) and (*v*_1_,*v*_2_) are connected if and only if (*u*_1_,*u*_2_) and (*v*_1_,*v*_2_) are different, *u*_1_,*v*_1_ are connected in *G*_1_ and *u*_2_,*v*_2_ are connected in *G*_2_ and for each *i*∈(1,…,5):
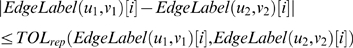
where *TOL_rep_* is a tolerance function defined as:
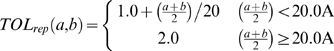
The function enforces an upper limit on the difference of two distances, which has been derived from the analysis of a set of protein-protein interfaces (unpublished).

After obtaining the product graph, maximal cliques are detected [33]. The cliques in the product graph correspond to aligned representatives. Only the largest alignments of representatives are consider in the following step.

#### Extending aligned representatives to NCIVs

Up to this stage, the alignment consists of aligned representatives of NCIV clusters. In this step these aligned representatives are used as “anchors” for deriving the alignment between the original sets of NCIVs.

First, in an *expanding* procedure, two NCIVs are matched if i) they are of the same type, ii) they have similar orientations (the angle between them is at most 45°) after the transformation based on the superposition of the anchors, and iii) they have similar distances to the anchors. A tolerance function for distance difference is defined as:
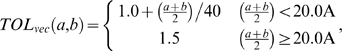
where *a* and *b* are the distances to be compared. *TOL_vec_* is more restrictive than *TOL_rep_*, as it is applied to actual NCIVs instead of representatives.

After finding all the potential alignments of NCIVs, a *filtering* procedure is performed. A pair of aligned NCIVs found in the *expanding* procedure is discarded if the difference of their distances to any other pair of aligned NCIVs exceeds the tolerance defined in *TOL_vec_*.

The resulting matched NCIVs replace the aligned representatives as new anchors, and the *expanding* and *filtering* procedures are repeated. Newly found matches of NCIVs are added to the anchors, until no more NCIVs can be matched in the *expanding* procedure. All resulting alignments of NCIVs are sorted according to alignment size (number of matched NCIVs). Only the largest alignments are reported. Alignments with a size below 90% of the largest one are discarded.

#### Availability

The source code of Galinter is available upon request from the authors.

### Comparison of alignments

#### Pilot Dataset

We have applied Galinter to the pilot dataset which was used for testing I2I-SiteEngine [17]. This dataset consists of 64 protein-protein interfaces clustered into 22 groups according to I2I-SiteEngine alignment results (see Figure S2 in the supplement). It is composed of a variety of protein complexes, including antigen-antibody, protease-inhibitor, protein-peptide, and protein-protein dimers. There are both homo- and hetero-dimers in the dataset. We excluded eight singleton groups from the dataset. This analysis is restricted to the remaining 14 non-singleton groups.

For any pair of complexes to be compared, if at least one subunit of one complex is homologous to at least one subunit of the other complex, then the two complexes are labeled as S/D-homologous (single- or double-sided homologous). Otherwise the two complexes are labeled as non-homologous. Two subunit structures are considered to be homologous if they belong to the same superfamily in SCOP [34]. In nine of the 14 groups, all complexes are S/D-homologous to each other within the group. The remaining groups also contain some complexes not related by homology. See Figure S3, S4, S5 in the supplement for more details.

#### Comparing Galinter to I2I-SiteEngine and DaliLite

On the pilot dataset, Galinter alignments were compared to the alignments generated by the I2I-SiteEngine interface comparison method. I2I-SiteEngine matches chemical functional groups and associated residues at the binding sites of different interfaces. In addition, we compared the results of both Galinter and I2I-SiteEngine to alignments based on backbone structure, generated with DaliLite [35]. Using DaliLite, subunit structures are compared individually at both sides of interfaces. A subsequent alignment of interface residues can be derived based on the most significant DaliLite alignment of subunit structures as detailed in Figure S6 in the supplement.

#### Assessing the agreement of the results

In this work, we define interface residues as those which contain at least one interface atom, where interface atoms are the atoms involved in interface NCIVs. We compared the alignment of interfaces from the different methods (Galinter, I2I-SiteEngine, and DaliLite) by examining the deviation of C_α_ atom coordinates of interface residues after corresponding transformations. Given two interface residue sets *I_1_* and *I_2_* and two alignment methods *M_a_* and *M_b_*, let *I_2a_* correspond to the transformed set *I_2_* according to the optimal superposition based on the alignment from method *M_a_*. Analogously, *I_2_* is transformed to *I_2b_* based on the alignment from method *M_b_*. Then, the root-mean-square deviation (RMSD) for all C_α_ atoms of interface residues in *I_2a_* and *I_2b_* is calculated to assess the agreement between the two methods *M_a_* and *M_b_*. This measure is defined as irRMSD (interface residue RMSD). See Figure S7 in the supplement for an illustration of the calculation of irRMSD.

## Results

To assess whether Galinter produces valid interface alignments, we compared the results of Galinter to the alignments generated by other approaches. One of these approaches aligns functional chemical groups at interfaces (I2I-SiteEngine) and the other approach aligns backbone structures (DaliLite).

In the second part of this section, we present the application of Galinter to three mimicry cases, for which the interfaces have been manually compared before.

### Application results on the pilot dataset

#### Comparison between Galinter, I2I-SiteEngine, and DaliLite

We have applied Galinter to every pair of interfaces within each of the 14 groups from the pilot dataset. There are 240 comparisons in total. The mean run time is 138.5 seconds (median run time 71.5 seconds) on a normal desktop (3.0 GHz CPU, 1GB memory) for these comparisons. The alignment results are compared to those of I2I-SiteEngine and DaliLite. The extent of agreement is measured using irRMSD values as described in section “***Assessing the agreement of the results***”.

I2I-SiteEngine compares interfaces by aligning the functional groups at binding sites, instead of aligning molecular interactions within the interface like Galinter. Galinter and I2I-SiteEngine can be regarded as complementary approaches as they use different properties to compare interfaces.

Backbone structure comparison methods like DaliLite can be used to generate interface alignments indirectly. These alignments are indirect in the sense that they do not take the structural similarities of the interfaces into account explicitly. When the interaction orientations of subunits are conserved between S/D-homologous complexes, these indirect alignments provide a coarse way of validating alignments from direct methods like Galinter and I2I-SiteEngine. The alignments based on backbone structures are expected to agree with explicit alignments of non-covalent interactions within the interfaces to some extent but not necessarily to match them.

**Figure 2:**
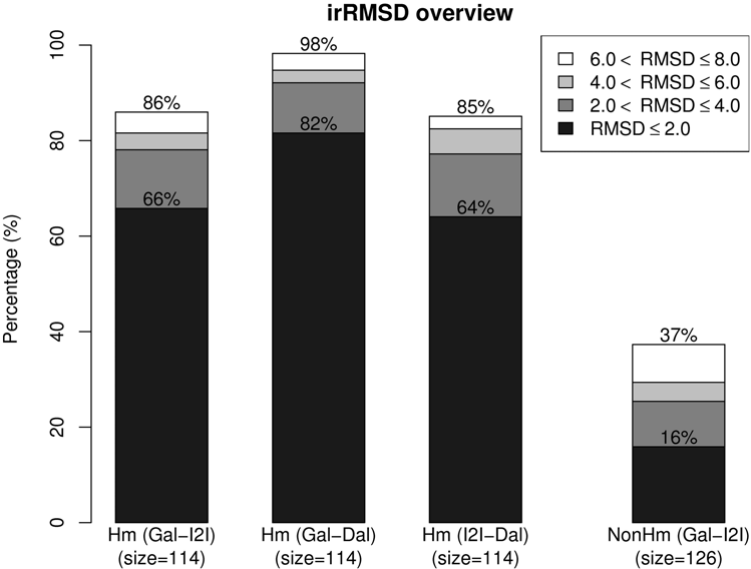
Overview of irRMSD values for pairwise comparison of protein-protein interfaces. Most interfaces for non-homologous complexes cannot be compared using backbone alignment method. Thus for the alignments of non-homologous complex interfaces, only an overview of irRMSD values for the comparison between Galinter and I2I-SiteEngine are shown. (Hm: S/D-homologous; NonHm: non-homologous; Gal: Galinter; I2I: I2I-SiteEngine; Dal: DaliLite)

Figure 2 provides a summary of the irRMSD values obtained in the analysis. All pairwise comparisons of interfaces are separated into two groups according to whether the corresponding complexes are S/D-homologous or non-homologous. Of the 240 pairs of interfaces compared, 114 are S/D-homologous and the remaining 126 pairs are non-homologous. For the alignments of non-homologous interfaces, only irRMSD values for the comparison between Galinter and I2I-SiteEngine are shown, because most non-homologous interfaces cannot be aligned using DaliLite as there is no backbone structure similarity between the respective protein complexes.

Figure 2 shows that for S/D-homologous interfaces, Galinter alignments usually agree with I2I-SiteEngine alignments. The alignments are similar (irRMSD≤2 Å) for 66% of the cases. Galinter and I2I-SiteEngine both produce similar alignments to DaliLite if the interfaces are S/D-homologous. But the agreement between Galinter and DaliLite is higher, in general, than that between I2I-SiteEngine and DaliLite. For non-homologous interfaces, Galinter and I2I-SiteEngine generate very different alignments. Less than 40% of the 126 comparisons have irRMSD values below 8 Å. The supplement contains the results for each comparison (Figure S3, S4, S5).

We have explored possible causes for the disagreements between the alignments of different methods. For non-homologous interfaces, most of the disagreements are observed in groups 19 and 5. Group 19 consists of coiled-coil interfaces. More than a single solution is expected for the alignment of these repetitive structures. Therefore it is not surprising that the alignments from different methods disagree. In general, the alignments of both methods result in reasonable superimposition of the helix backbones. Nevertheless, visual inspection reveals that for some of these pairs one of the methods generates better superposition of the interacting helices. Galinter produces better superposition in five pairs (1ic2CD *vs.* 1gl2BC, 1ic2CD *vs.* 1gk4AB, 1gl2AB *vs.* 1gk4AB, 1gl2BC *vs.* 1gk4AB, 1gk4AB *vs.* 1if3AB), and I2I-SiteEngine in three cases (1ic2CD *vs.* 1if3AB, 1gl2AB *vs.* 1if3AB, 1gl2BC *vs.* 1if3AB). For example, in the comparison of 1gl2AB and 1gk4AB, chain B of 1gl2 has 16 helix turns and they are all superposed based on the Galinter alignment, while only 8 helix turns are superposed based on the I2I-SiteEngine alignment.

In group 5, there are relatively few similarities between the subunits from different complexes. There seems to be no obvious alignment solution in terms of either structure or evolution. The only evident common feature in these interfaces is that they include two interacting β-strands. The assessment of the results in this group is thus challenging. Bearing this in mind, we have investigated the quality of the results by visual inspection of the superposition of the two strands at the interfaces. We have found that for 15 pairs Galinter provides better superposition of the interface β-strands, and for five pairs I2I-SiteEngines leads to better superposition of these strands.

The disagreements between Galinter and I2I-SiteEngine for S/D-homologous interfaces arise mainly from group 10, and also to a lesser extent, from the smaller group 4. Interestingly, for these two groups, the Galinter alignments agree with those based on DaliLite.

In general, the three methods agree to a large extent, especially when the interfaces are related by homology. Nevertheless, it is not surprising to observe disagreements in the non-homologous groups, considering both that Galinter and I2I-SiteEngine are based on different interface properties and that there are no unique solutions in these groups.

#### Contribution of different types of non-covalent interactions to the alignment

The current implementation of Galinter aligns vdW interactions and hydrogen bonds at interfaces. However, there are other types of non-covalent atomic interactions, especially electrostatic interactions between positively and negatively charged atoms. Thus we have explored the contribution of short-range electrostatic interactions to the alignment of protein-protein interfaces. Using a definition by Xu *et al.* [36], we have identified fewer than three short-range electrostatic interactions on average for each of the 64 interfaces in the pilot dataset used in the manuscript. This is only 1% of the number of vdW interactions. In addition, we have re-ranked the alignment results by assigning the larger weight of 3 to short-range electrostatic interactions (versus a weight of 1 to vdW interactions and hydrogen bonds). Except for four cases (1okvBE vs. 1okuBF, 10gsAB vs. 1axdAB, 1axdAB vs. 10gsAB, 1g0uOP vs. 1iruFG), the top-ranking alignments for the pilot dataset remain the same. Even for these four cases, the new results exhibit considerable similarity to the original alignments (half or more of the aligned NCIVs are the same).

These results indicate that the current method seems to be robust with respect to different weighting of the various types of interactions. Nevertheless, a thorough investigation is required on how to weight different types of non-covalent interactions for interface alignment, which will be the focus of future work.

### Analysis of mimicry cases

Protein mimicry is relevant in the design of protein inhibitors. These inhibitors are frequently designed such that their binding mode is similar to that of a wild-type protein-protein interaction. Their development process is expected to benefit from detailed comparisons of the non-covalent interactions. We have applied Galinter to studying the protein-protein interaction mechanisms of three cases of protein mimicry: i) Chymotrypsin and subtilisin interact with the same type of inhibitors, an example of convergent evolution [37]; ii) A scorpion-toxin derived compound (CD4M33-F23) mimics CD4 in complex with gp120, a mimicry case relevant to HIV therapy [38]; iii) A non-peptidic compound SP4206 mimics IL-2Rα in binding to IL-2 [39].

In each of these three cases, the subunits are homologous only on one side of the interface. In the third case, one of the interacting partners is not even a protein.

#### Comparison of two protease-inhibitor interfaces

The Ser–His–Asp catalytic triad present in many proteases has been intensively analyzed [40], [41]. This catalytic triad occurs in several protein families which are non-homologous, and therefore have no significant backbone structure similarity [42]. Specifically, the trypsin-like serine proteases chymotrypsin and subtilisin belong to different SCOP superfamilies (*sccs* codes: b.47.1.2 and c.41.1.1, respectively). Although they lack obvious sequence or structure similarity, they have been found to share as many as three inhibitors [13].

**Figure 3:**
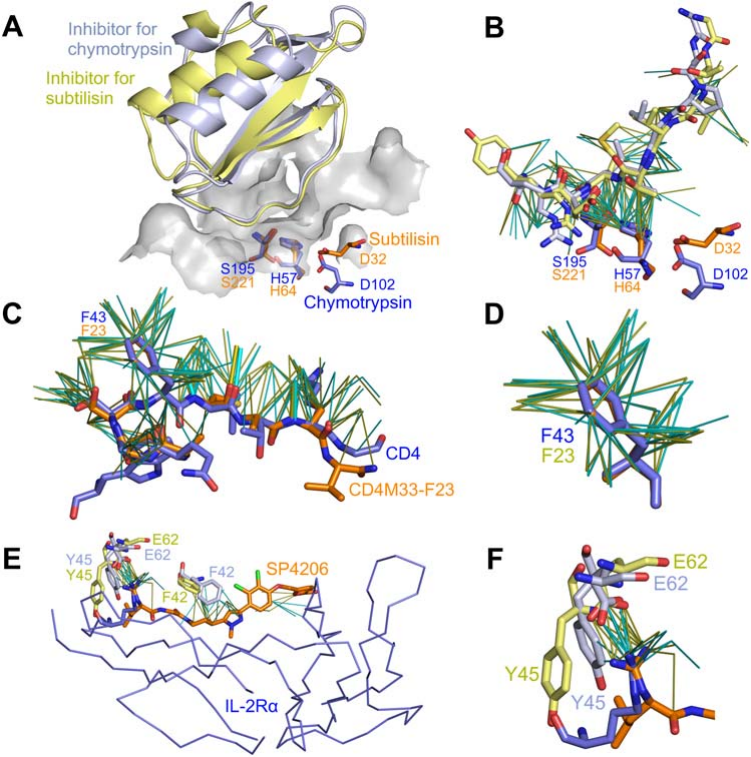
Analysis of mimicry cases. Every example is shown with two representations in the same orientation. In all representations, the homologous side is in light blue and light yellow at the top, the mimic side is shown in dark blue and orange at the bottom. NCIVs at interfaces are shown as thin lines. A) Superposed inhibitors and catalytic triads for chymotrypsin (1acb) and subtilisin (1lw6) according to the Galinter alignment. The inhibitor for Chymotrypsin is shown in light blue and the inhibitor for subtilisin is shown in light yellow. The catalytic triads of chymotrypsin and subtilisin are shown as sticks in dark blue and orange, respectively. The chymotrypsin binding site is shown as a gray surface. B) Superposed NCIVs for chymotrypsin/inhibitor interface (1acbEI) and subtilisin/inhibitor interface (1lw6EI) according to the Galinter alignment. Only matched NCIVs are shown. Chymotrypsin/inhibitor NCIVs are shown in cyan, and subtilisin/inhibitor NCIVs are shown in yellow. C) Superposed NCIVs for CD4/gp120 interface (1rzjCG) and CD4M33-F23/gp120 interface (1yymMG) according to the Galinter alignment. CD4 is shown in dark blue and CD4M33-F23 is in orange. Only matched NCIVs are shown. CD4/gp120 NCIVs are shown in cyan, and CD4M33-F23/gp120 NCIVs are in yellow. Hydrogen bonds are shown as thick lines. D) An enlarged view of the matched NCIVs involving the hot spot phenylalanines. E) Superposed NCIVs according to the Galinter alignment of IL-2Rα/IL-2 interface (1z92BA) in dark and light blue, and of SP4206/IL-2 interface (1py2_A) in orange and light yellow. Only matched NCIVs are shown. IL-2Rα/IL-2 NCIVs are shown in cyan, SP4206/IL-2 NCIVs are in yellow. The hot spot residues Phe42, Tyr45, and Glu62 in IL-2 are shown as sticks. F) An enlarged view of the *mimic spot* around residue Glu62 in IL-2. PyMOL [46] has been used to produce the representations.

We have analyzed the interactions formed between chymotrypsin and leech proteinase inhibitor eglin c (PDB code: 1acb, chains E and I), and subtilisin with chymotrypsin inhibitor 2 (PDB code: 1lw6, chains E and I). The two protease inhibitors have similar backbone structures and belong to the same SCOP family (b.40.1.1). The two interfaces contain 299 and 332 NCIVs, respectively. The longest Galinter alignment consists of 117 aligned NCIVs, and the results are visualized in Figure 3A and 3B. According to this alignment, the two catalytic triads are superposed with an RMSD of 0.5 Å (Figure 3A). The RMSD is computed for the overall functional template atoms of the catalytic triads as defined in Wallace *et al.* [37]. Figure 3B displays superposed NCIVs according to Galinter at the two interfaces. It is noticeable that the NCIVs involving the catalytic serine and histidine residues are well conserved.

We have also compared the two interfaces based on inhibitor backbone alignment. First the inhibitor structures of the two complexes have been aligned using DaliLite. Then the two proteases have been superposed accordingly. This way an alignment of the interfaces is obtained indirectly. This indirect alignment agrees with the Galinter alignment to a considerable extent (irRMSD = 2.7 Å). Based on this indirect alignment, the RMSD for the overall functional template atoms of the catalytic triads is much larger than the one obtained based on the Galinter alignment (2.2 Å *vs.* 0.5 Å). This is not surprising given that these catalytic residues are not used by DaliLite when computing the alignment. Meanwhile, these results also indicate that to compare protein-protein interfaces, an explicit interface alignment approach is more adequate than an approach based on backbone structure.

#### Analysis of a scorpion-toxin derived mimic of CD4 in complex with gp120

In order for HIV to infect host cells, the HIV envelope glycoprotein gp120 binds CD4 receptors located on the target cell surfaces. The CD4 binding site for gp120 has been engineered onto a scorpion-toxin protein, resulting in CD4M33-F23. Recently, the mimicked interaction of CD4M33-F23 in complex with gp120 has been investigated in detail and compared to the native complex structure of CD4 and gp120 [38]. In particular, Huang and colleagues analyzed the difference distance matrix between the two complexes for gp120 residues surrounding the hot spot residue Phe43 of CD4.

We have compared the natural complex interfaces (PDB code: 1rzj, chains C and G) and mimicry interface (PDB code: 1yym, chains M and G) using Galinter. The numbers of NCIVs are 364 for 1rzjCG and 166 for 1yymMG. In spite of the lack of similarities between the overall folds of CD4 and CD4M33-F23, about 80% (133 NCIVs) of the NCIVs at the CD4M33-F23/gp120 interface have been aligned to those at the CD4/gp120 interface. In addition, three of the four interface hydrogen bonds aligned as described in Huang *et al.* [38] are also aligned in the same way by Galinter (Figure 3C).

We have also observed that the hot spot residue Phe43 in CD4 (or equivalent residue Phe23 in CD4M33-F23) is in contact with eight residues of gp120 (Asp368, Glu370, Ile371, Asn425, Met426, Trp427, Gly473, and Met475) via 46 vdW interactions of 133 total aligned NCIVs in both interfaces. All these NCIVs have been aligned by Galinter successfully (Figure 3D).

#### SP4206 mimic of IL-2Rα in binding to IL-2

Thanos *et al.* [43] have published the structure of the small compound SP4206 binding to an IL-2 cytokine, which in turn blocks the natural interaction of IL-2 and its receptor IL-2Rα. Interestingly, although the interface size of SP4206 and IL-2 is only half as large as that between IL-2Rα and IL-2, SP4206 and IL-2Rα bind to IL-2 with similar affinities. Thanos and colleagues have discovered that this is mainly because SP4206 utilizes the same hot spot residues as IL-2Rα when interacting with IL-2 [39].

We have compared the interface of IL-2Rα and IL-2 (PDB code: 1z92, chains B and A), with the interface formed between SP4206 and IL-2 (PDB code: 1py2, FRH and chain A) using Galinter. The protocol has been slightly modified in order to identify hydrogen bonds between a non-peptidic molecule and a protein. HBPLUS [29] has been used to infer hydrogen bonds within the interface between SP4206 and IL-2. We have identified 330 NCIVs for IL-2Rα/IL-2 interface, and 176 NCIVs for SP4206/IL-2 interface. The alignment results are shown in Figure 3E. Only a small number (35) of the interface NCIVs are aligned by Galinter. We have found that the main reason for this relatively short alignment is that the IL-2 binding sites adopt different conformations when binding the two partners. Particularly, two of the three hot spot residues on IL-2 binding sites (Phe42 and Tyr45) adopt different side chain formations in the interfaces. Only Glu62 is structurally conserved. In IL-2Rα/IL-2, this residue forms salt bridges with the guanido group of residue Arg36 in IL-2Rα. In SP4206/IL-2, we observe similar interactions between the carboxyl group of IL-2 Glu62 and the guanido group in SP4206 [39]. Galinter correctly identifies these conserved interactions (see Figure 3F). Apparently the similarities are not uniformly distributed along the interfaces. It is noticeable that in proximity of residue Glu62 the NCIVs are conserved, while NCIVs are only sparsely aligned in the rest of the interfaces. We label this conserved interface region a *mimic spot*, in analogy to the concept of hot spot, which refers to residues contributing to a large fraction of the binding energy [44].

#### Comparison to I2I-SiteEngine results

We have applied I2I-SiteEngine to align the three pairs of mimicry interfaces. In the case of the two protease-inhibitor interfaces, I2I-SiteEngine generates a similar alignment to Galinter with an irRMSD of 1.0 Å. The RMSD for the overall functional template atoms of the two catalytic triads is worse than that calculated based on Galinter alignment (1.1 Å *vs.* 0.5 Å). In addition, the RMSD for the two inhibitors is 4.2 Å which is higher than that obtained based on Galinter result (2.9 Å). For the second mimicry case, the I2I-SiteEngine alignment agrees with the Galinter result, with an irRMSD of only 0.4 Å. In the third mimicry case, one of the subunits participating in the interaction is a non-peptidic molecule (SP4206) and we could not obtain I2I-SiteEngine alignment. I2I-SiteEngine is only applicable to interfaces consisting of interacting proteins as it relies on the definition of functional groups of amino acids. This definition is not available for non-peptidic molecules. In this respect Galinter is more general than I2I-SiteEngine as it can also be applied to interfaces involving non-peptidic molecules.

## Discussion

We have presented Galinter, a novel method for explicitly comparing interfaces based on the geometry and type of non-covalent interactions. The proposed method complements existing approaches to the analysis of protein-protein interfaces. The method was applied to the pilot dataset [17] and compared to an interface alignment method and to a backbone structure alignment method. It is reassuring that for S/D-homologous complexes we have obtained consistent results with the three methods. For non-homologous complexes, Galinter provides alternative solutions that tend to match common secondary structure elements at the interfaces. In addition, Galinter has been applied to comparing mimicry examples, and the results are consistent with previous human-curated analyses. The results also suggest that Galinter has the potential of assisting in the design of interaction inhibitors. In addition, as shown in the IL-2Rα mimicry example, Galinter is more general than existing approaches as it can compare interfaces in which non-peptidic molecules are involved.

Currently, the final Galinter alignments of NCIVs are ranked by their size in terms of the number of involved NCIVs, but a more comprehensive scoring function for alignments is desirable. Geometric and chemical similarity of matched NCIVs should be taken into account when computing alignment scores. Ideally such a scoring function should provide a statistical significance value for each alignment as well. This will be the focus of future work.

We have demonstrated the application of Galinter to the comparison of protein-protein interfaces, and also to the comparison of a protein-protein interface with an interface between a protein and a non-peptidic molecule (ligand). Galinter may also be applied for comparing protein-ligand to protein-ligand interfaces. But for this purpose the approach needs to be further tested. In addition, the interfaces in the current work have been defined between different polypeptide chains. However, the method is also applicable to the comparison of interfaces formed between protein domains along the same chain.

In the comparison of SP4206/IL-2 and IL-2Rα/IL-2, we have observed a non-uniform distribution of conserved NCIVs throughout the two interfaces. The NCIVs involving residue Arg36 on IL-2Rα and its counterpart guanido group on SP4206 are highly conserved. Similar results have also been observed in the first and second case studies. In the case of the protease/inhibitor interfaces, a large fraction of aligned NCIVs involve the two catalytic residues serine and histidine. At CD4/gp120 and CD4M33-F23/gp120 interfaces, Phe43 in CD4 and Phe23 in CD4M33-F23, respectively, form 46 NCIVs with eight surrounding residues (see Figure 3D). All these NCIVs are aligned and account for 35% of the final alignment. We call these conserved interface regions *mimic spots*. We plan to extend the functionality of Galinter to the automatic detection of conserved interface regions, as in the case of *mimic spots*. The relationship between conserved interface regions, *mimic spots* and hot spots is another interesting topic deserving further study. Recent results indicate that conserved regions and hot spots overlap to a considerable extent [45].

## Supporting Information

Figure S1 Geometric criteria for identifying hydrogen bonds.
(1.17 MB TIF)

Figure S2 Pilot dataset.
(0.60 MB TIF)

Figure S3 Galinter vs. I2I-SiteEngine. Heat maps for irRMSD values of interface residues. Only the 14 non-singleton groups in the pilot dataset are shown. The heat maps are sorted by size. The columns and rows for each heat map represent interfaces identified by their PDB code and chain names constituting the interfaces. The diagonal grids of all heat maps have been left blank. For S/D-homologous complexes, S/D-homology is indicated in corresponding grids by either a plus sign (+) for double-sided homology, or a minus sign (−) for single-sided homology. The heat maps have been produced using R (http://www.R-project.org).
(0.78 MB TIF)

Figure S4 Galinter vs. DaliLite. Heat maps for irRMSD values of interface residues. Only the 14 non-singleton groups in the pilot dataset are shown. The heat maps are sorted by size. The columns and rows for each heat map represent interfaces identified by their PDB code and chain names constituting the interfaces. The diagonal grids of all heat maps have been left blank. For S/D-homologous complexes, S/D-homology is indicated in corresponding grids by either a plus sign (+) for double-sided homology, or a minus sign (−) for single-sided homology. The heat maps have been produced using R (http://www.R-project.org).
(0.77 MB TIF)

Figure S5 I2I-SiteEngine vs. DaliLite. Heat maps for irRMSD values of interface residues. Only the 14 non-singleton groups in the pilot dataset are shown. The heat maps are sorted by size. The columns and rows for each heat map represent interfaces identified by their PDB code and chain names constituting the interfaces. The diagonal grids of all heat maps have been left blank. For S/D-homologous complexes, S/D-homology is indicated in corresponding grids by either a plus sign (+) for double-sided homology, or a minus sign (−) for single-sided homology. The heat maps have been produced using R (http://www.R-project.org).
(0.77 MB TIF)

Figure S6 Alignment of interfaces based on backbone structure. Using DaliLite, subunit structures are compared individually at both sides of interfaces. A subsequent alignment of interface residues can be derived based on the most significant DaliLite alignment of subunit structures.
(1.38 MB TIF)

Figure S7 Comparison of interface alignments using irRMSD measure. Given two interface residue sets *I*_1_ and *I*_2_ and two alignment methods *M*_a_ and *M*_b_, let *I*_2a_ correspond to the transformed *I*_2_ according to the optimal superposition based on the alignment from method *M*_a_. Analogously, *I*_2_ is transformed to *I*_2b_ based on the alignment from method *M*_b_. Then, the root-mean-square deviation (RMSD) for all C_α_ atoms of interface residues in *I*_2a_ and *I*_2b_ is calculated and reported as irRMSD to assess the agreement between the two methods. (NCIV: non-covalent interaction vector)
(1.30 MB TIF)
