# Supplementary figures and images for: Alignment of Non-Covalent Interactions at Protein-Protein Interfaces
Source: PLoS One. 2008 Apr 2;3(4):e1926. doi: 10.1371/journal.pone.0001926 (PMC2274958; doi:10.1371/journal.pone.0001926)

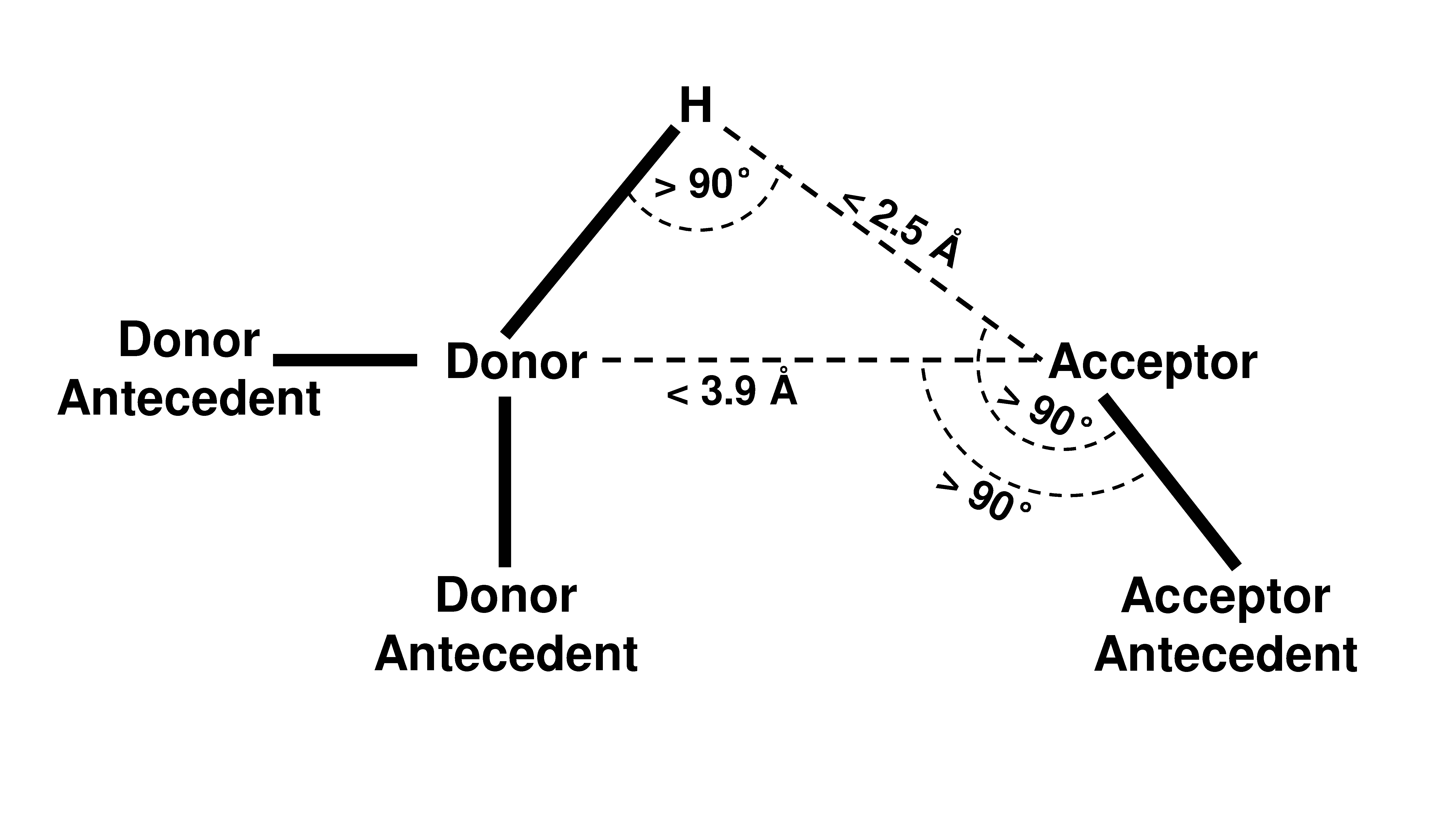

Supplement: Figure S1 — Geometric criteria for identifying hydrogen bonds. (1.17 MB TIF) [file pone.0001926.s001.tif]

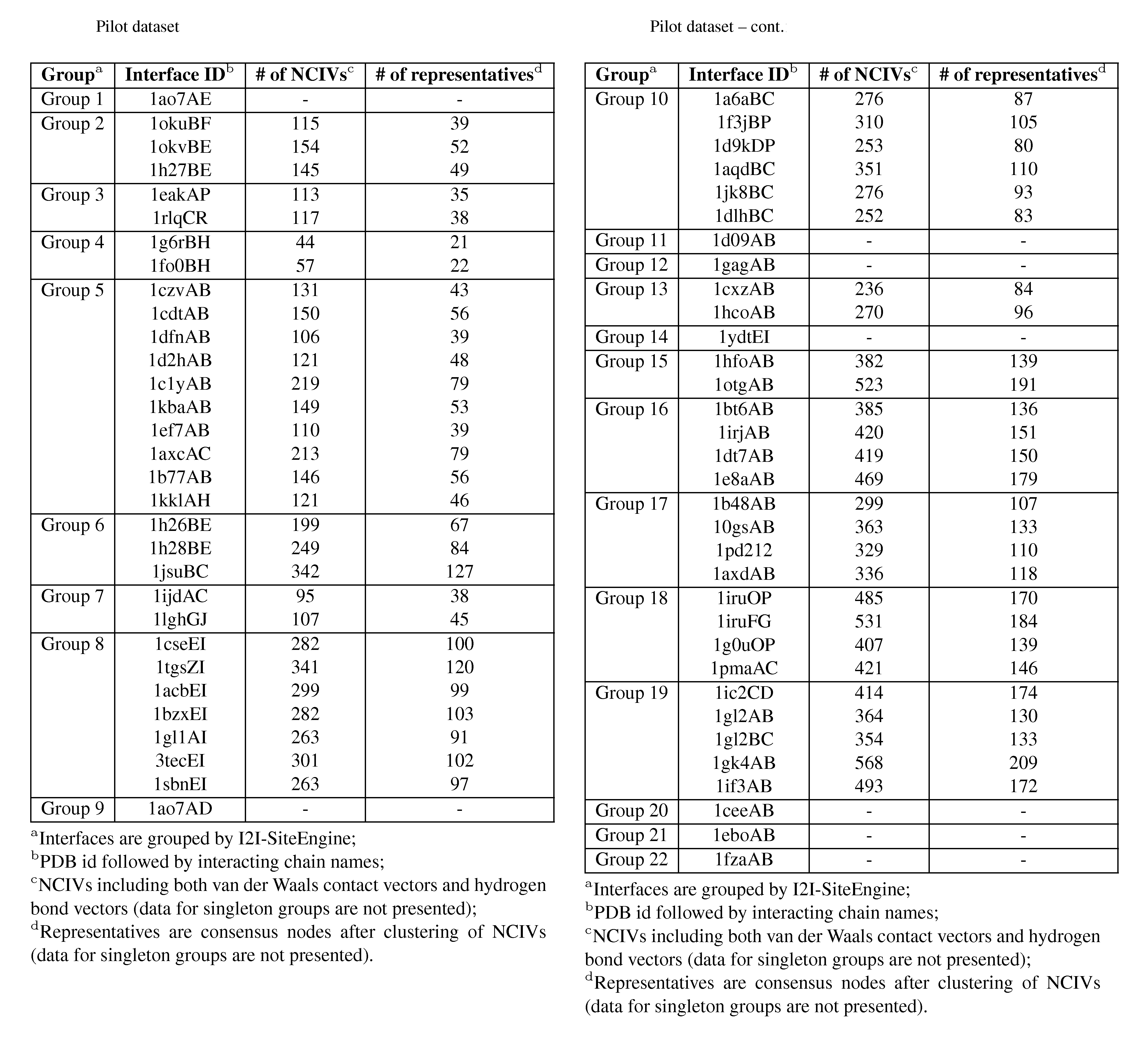

Supplement: Figure S2 — Pilot dataset. (0.60 MB TIF) [file pone.0001926.s002.tif]

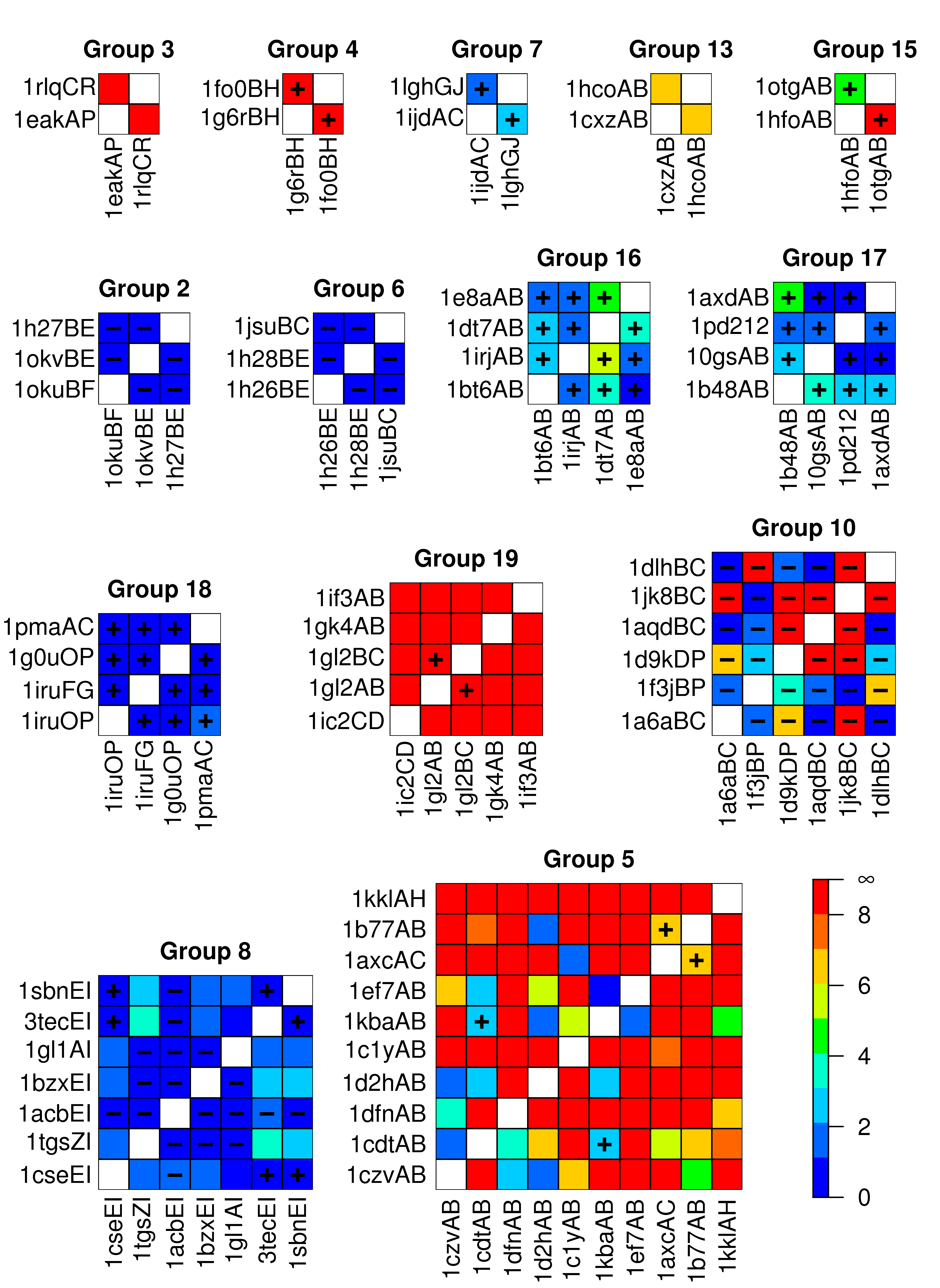

Supplement: Figure S3 — Galinter vs. I2I-SiteEngine. Heat maps for irRMSD values of interface residues. Only the 14 non-singleton groups in the pilot dataset are shown. The heat maps are sorted by size. The columns and rows for each heat map represent interfaces identified by their PDB code and chain names constituting the interfaces. The diagonal grids of all heat maps have been left blank. For S/D-homologous complexes, S/D-homology is indicated in corresponding grids by either a plus sign (+) for double-sided homology, or a minus sign (−) for single-sided homology. The heat maps have been produced using R (http://www.R-project.org). (0.78 MB TIF) [file pone.0001926.s003.tif]

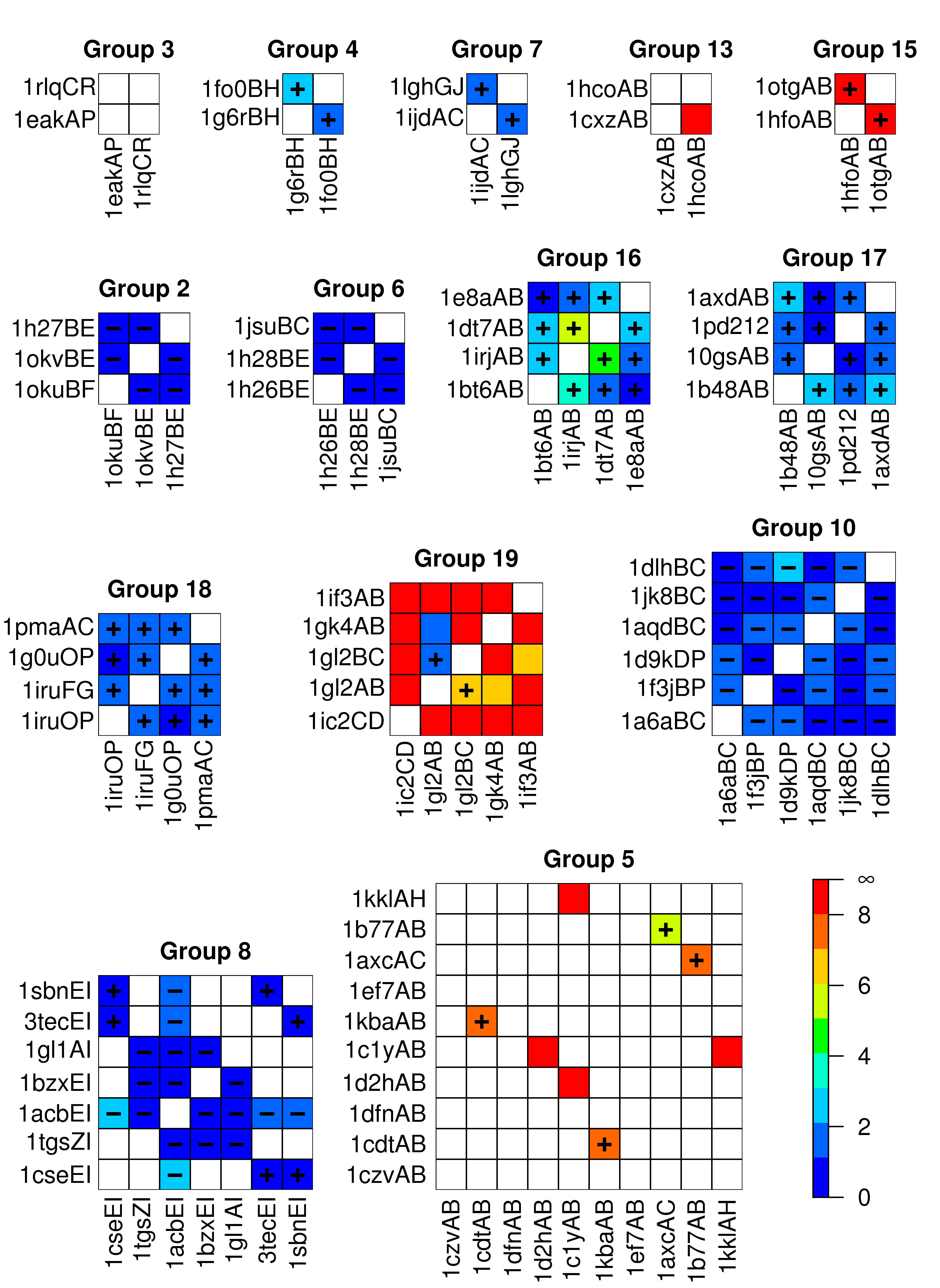

Supplement: Figure S4 — Galinter vs. DaliLite. Heat maps for irRMSD values of interface residues. Only the 14 non-singleton groups in the pilot dataset are shown. The heat maps are sorted by size. The columns and rows for each heat map represent interfaces identified by their PDB code and chain names constituting the interfaces. The diagonal grids of all heat maps have been left blank. For S/D-homologous complexes, S/D-homology is indicated in corresponding grids by either a plus sign (+) for double-sided homology, or a minus sign (−) for single-sided homology. The heat maps have been produced using R (http://www.R-project.org). (0.77 MB TIF) [file pone.0001926.s004.tif]

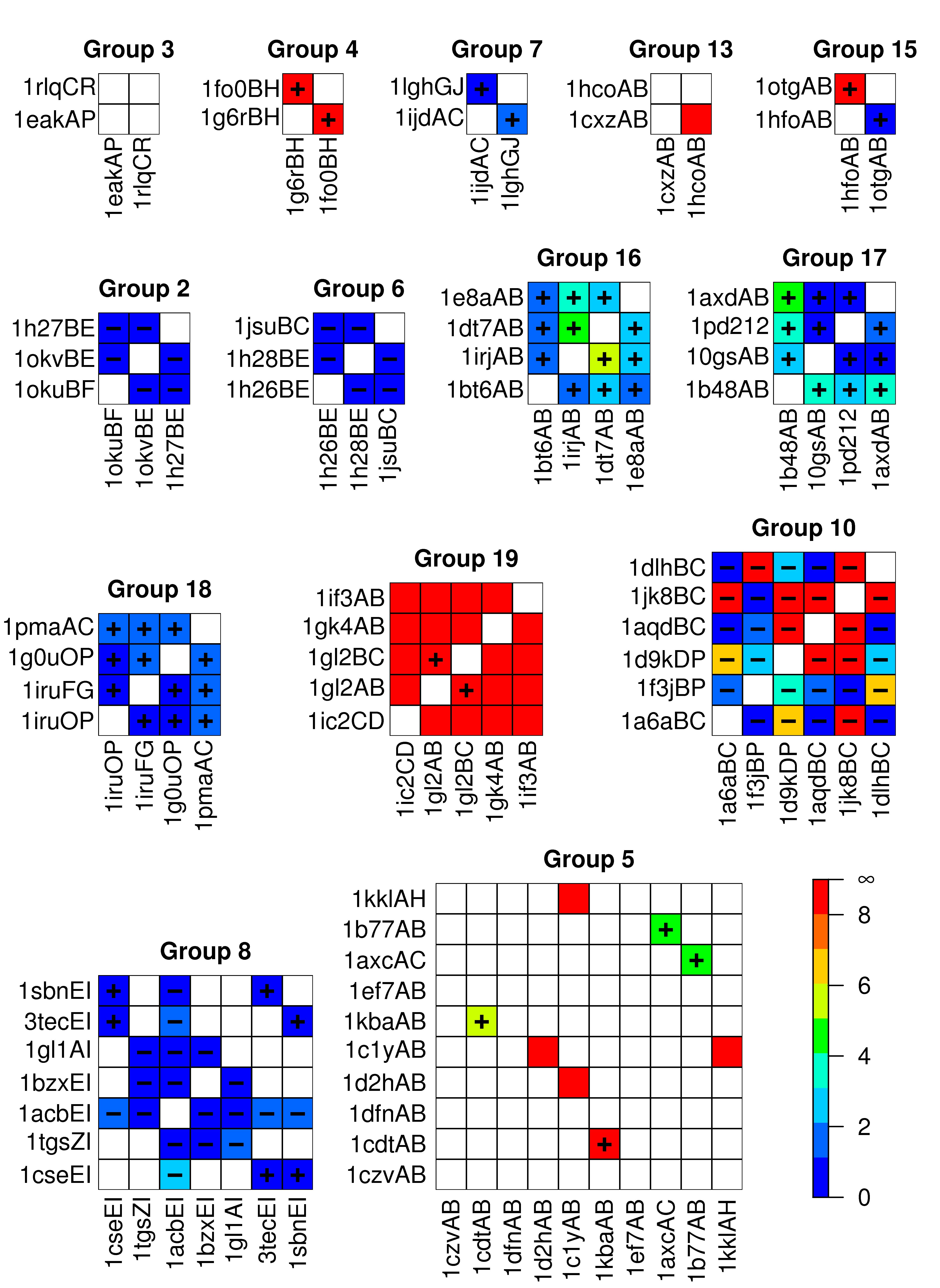

Supplement: Figure S5 — I2I-SiteEngine vs. DaliLite. Heat maps for irRMSD values of interface residues. Only the 14 non-singleton groups in the pilot dataset are shown. The heat maps are sorted by size. The columns and rows for each heat map represent interfaces identified by their PDB code and chain names constituting the interfaces. The diagonal grids of all heat maps have been left blank. For S/D-homologous complexes, S/D-homology is indicated in corresponding grids by either a plus sign (+) for double-sided homology, or a minus sign (−) for single-sided homology. The heat maps have been produced using R (http://www.R-project.org). (0.77 MB TIF) [file pone.0001926.s005.tif]

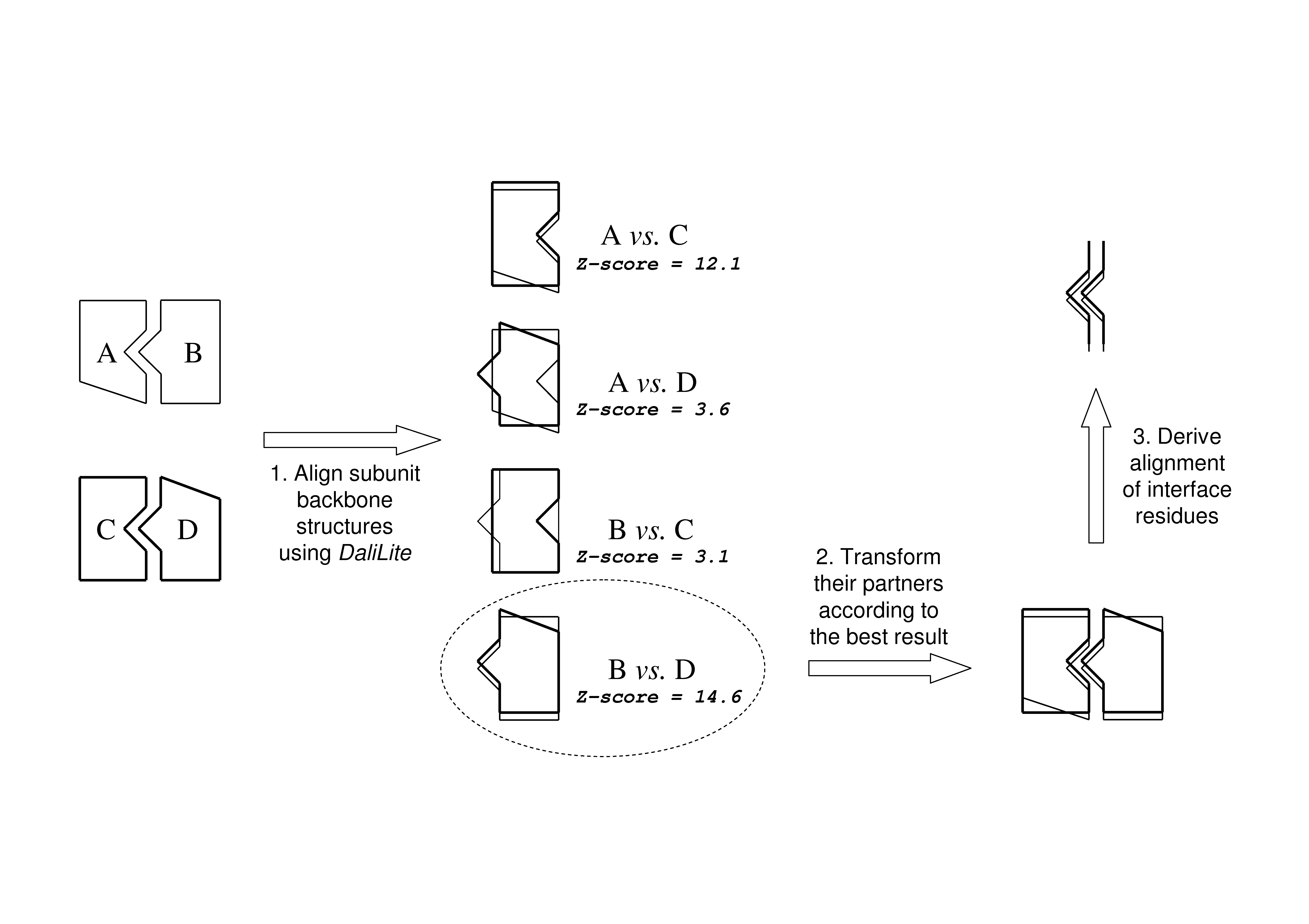

Supplement: Figure S6 — Alignment of interfaces based on backbone structure. Using DaliLite, subunit structures are compared individually at both sides of interfaces. A subsequent alignment of interface residues can be derived based on the most significant DaliLite alignment of subunit structures. (1.38 MB TIF) [file pone.0001926.s006.tif]

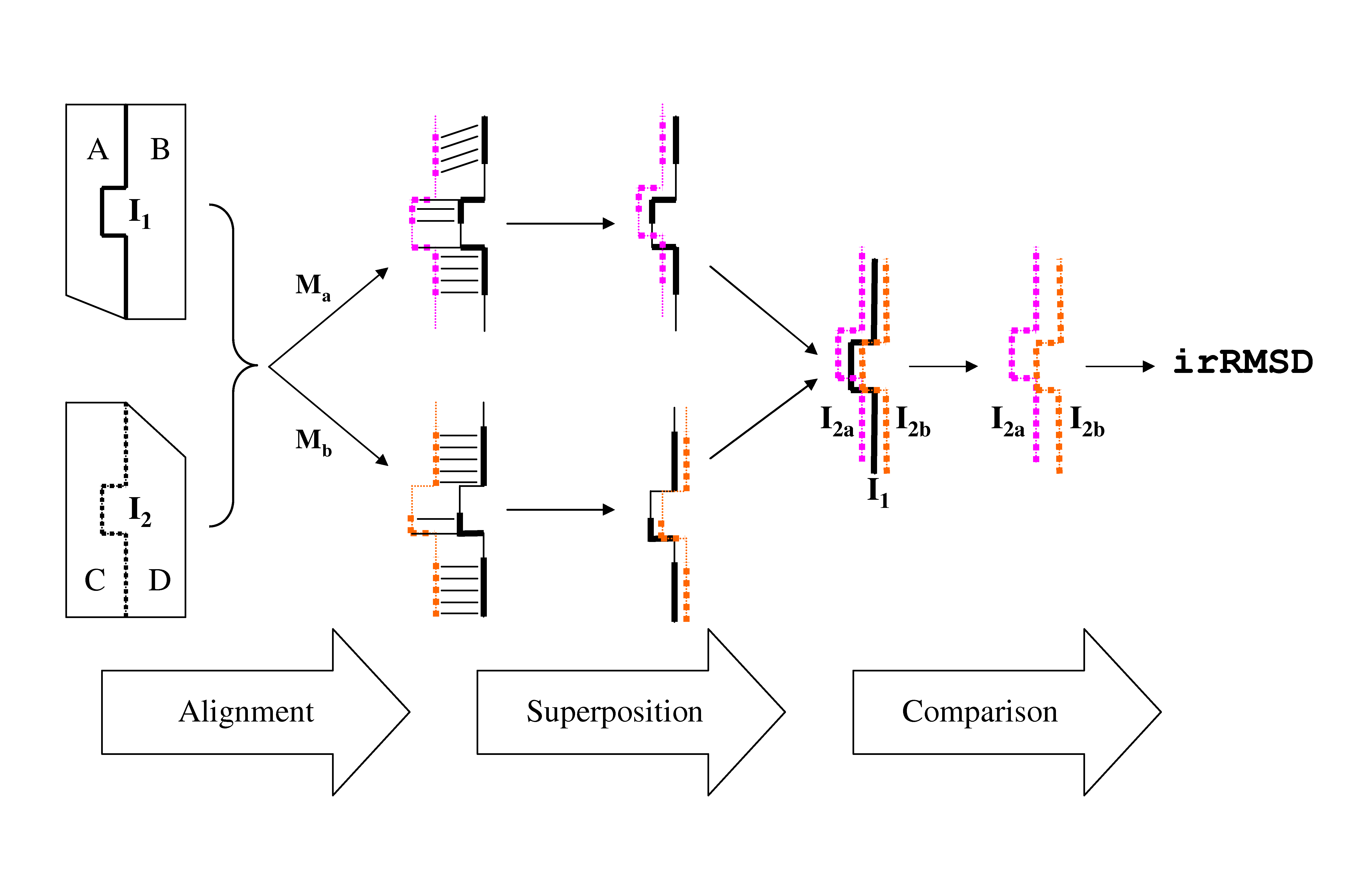

Supplement: Figure S7 — Comparison of interface alignments using irRMSD measure. Given two interface residue sets I 1 and I 2 and two alignment methods M a and M b, let I 2a correspond to the transformed I 2 according to the optimal superposition based on the alignment from method M a. Analogously, I 2 is transformed to I 2b based on the alignment from method M b. Then, the root-mean-square deviation (RMSD) for all Cα atoms of interface residues in I 2a and I 2b is calculated and reported as irRMSD to assess the agreement between the two methods. (NCIV: non-covalent interaction vector) (1.30 MB TIF) [file pone.0001926.s007.tif]
